# A Treatise on Diseases of the Eye

**Published:** 1843-06

**Authors:** 


Art. VII.—*A Treatise on Diseases of the Eye.* By William Lawrence, F. R. S., Surgeon Extraordinary to the Queen, Surgeon to St. Bartholomew's Hospital, &c., &c. From the last London edition. *With numerous additions and sixty-seven Illustrations.* By Isaac Hays, M. D., Surgeon to Will's Hospital, &c., &c.

This is a very beautiful book, and is, without doubt, the most splendid treatise on the subject in the language. We know of nothing equal to it, and we hope that it will meet with a wide circulation. Affections of the eye are too much neglected by the profession generally; the subject is very inadequately discussed in the majority of our schools, but one or two of which have a chair devoted to ophthalmic medicine and surgery; in all the others it is despatched in about a half-dozen lectures. In the west at least, these diseases are not infrequent—in certain districts, unless we mistake, they are very frequent; and a little more attention devoted to their study, might save the unfortunate from the tender mercies of the stationary or itinerant "eye-doctor," and from that direst of all calamities, *total and irremediable blindness.*

The following quotation from the very short preface of the American editor, will show what alterations and improvements have been made in the present edition.

"The present, is a reprint from the last London edition, which appeared in 1841, completely revised and greatly enlarged by the author; and to it considerable additions have been made by the Editor.

"Sixty-seven illustrations are introduced, many of them from original drawings, the whole engraved by Mr. R. S. Gilbert.

"Several subjects omitted in the original, are treated of; in supplying which, as well as on other occasions, free use has been made of the valuable Treatise of Mr. McKenzie. The catoptric examination of the eye, and its value as a means of diagnosis; the recent investigations into the structures of the orbit, &c., are all fully considered."

The reader must not suppose from the modest manner in which the editor thus speaks of his own labors that they are few or unimportant; they contain, what is of no small value, "the results of his experience in relation to the treatment of most of the important diseases, derived from more than twenty years devotion to the subject, during all which period he has been attached to some public institution for the treatment of Diseases of the Eye." C.

